# Zika Virus Non-structural Protein 4A Blocks the RLR-MAVS Signaling

**DOI:** 10.3389/fmicb.2018.01350

**Published:** 2018-06-25

**Authors:** Jinzhu Ma, Harshada Ketkar, Tingting Geng, Emily Lo, Leilei Wang, Juemin Xi, Qiangming Sun, Zhanbo Zhu, Yudong Cui, Long Yang, Penghua Wang

**Affiliations:** ^1^College of Life Science and Technology, Heilongjiang Bayi Agricultural University, Daqing, China; ^2^Department of Microbiology and Immunology, New York Medical College, Valhalla, NY, United States; ^3^Department of Obstetrics and Gynecology, Shengjing Hospital, China Medical University, Shenyang, China; ^4^Institute of Medical Biology, Chinese Academy of Medical Sciences, Peking Union Medical College, Kunming, China

**Keywords:** flavivirus, Zika, non-structural protein 4A, NS4A, RIG-I like receptors, RLR

## Abstract

Flaviviruses have evolved complex mechanisms to evade the mammalian host immune systems including the RIG-I (retinoic acid-inducible gene I) like receptor (RLR) signaling. Zika virus (ZIKV) is a re-emerging flavivirus that is associated with severe neonatal microcephaly and adult Guillain-Barre syndrome. However, the molecular mechanisms underlying ZIKV pathogenesis remain poorly defined. Here we report that ZIKV non-structural protein 4A (NS4A) impairs the RLR-mitochondrial antiviral-signaling protein (MAVS) interaction and subsequent induction of antiviral immune responses. In human trophoblasts, both RIG-I and melanoma differentiation-associated protein 5 (MDA5) contribute to type I interferon (IFN) induction and control ZIKV replication. Type I IFN induction by ZIKV is almost completely abolished in *MAVS*^-/-^ cells. NS4A represses RLR-, but not Toll-like receptor-mediated immune responses. NS4A specifically binds the N-terminal caspase activation and recruitment domain (CARD) of MAVS and thus blocks its accessibility by RLRs. Our study provides in-depth understanding of the molecular mechanisms of immune evasion by ZIKV and its pathogenesis.

## Introduction

Zika virus (ZIKV) is a re-emerging *Flaviviridae* member that was first identified in a sentinel rhesus monkey in the Zika Forest of Uganda in 1947 (Dick et al., 1952). ZIKV then spread to Asia and more recently the Central and South America (Cao-Lormeau and Musso, 2014; Colon-Gonzalez et al., 2017; Fu et al., 2017; Perry et al., 2017). Increasing evidence suggests that ZIKV infection is responsible for severe neurological complications such as neonatal microcephaly, adult Guillain-Barre syndrome, and maculopathy (Oehler et al., 2014; Cao-Lormeau et al., 2016; Miranda-Filho Dde et al., 2016; Petersen et al., 2016). Although intensive efforts are being invested, unfortunately no vaccines or therapeutics are available to prevent and treat ZIKV infection to date (Fauci and Morens, 2016).

Type I interferons (IFNs) are a critical host defense system against ZIKV infection and are initiated primarily by retinoic acid-inducible gene I (RIG-I)-like receptors (RLRs), which sense cytoplasmic dsRNA (Loo and Gale, 2011). The RLR family has 3 members, RIG-I, melanoma differentiation-associated gene 5 (MDA5), and Laboratory of Genetics and Physiology 2 (LGP2). The function of MDA5 and RIG-I in RNA virus infection has been well established. Once bound by viral RNA, RIG-I/MDA5 will undergo conformational change and expose its N-terminal caspase-recruitment domains (CARD) which bind the CARD of mitochondrial antiviral-signaling protein (MAVS). MAVS subsequently recruits TRAF3 and TRAF6 to its C-terminus and activates downstream signaling proteins such as TBK1, NF-κB, IRF3, and IRF7, up-regulating expression of type I interferon (IFN) as well as inflammatory cytokines and chemokines (Seth et al., 2006; Sun et al., 2010).

The flaviviral genome is a single stranded, positive-sense RNA encoding an envelope protein (E) and a pre-membrane/membrane (PrM/M) that make up the viral envelope together with a lipid bilayer as well as seven non-structural proteins (NS1, NS2A, NS2B, NS3, NS4A, NS4B, and NS5) that are required for viral replication and are involved in immune evasion (Ngono and Shresta, 2018). The ability of many medically important flaviviruses to interfere with the RLR signaling and type I IFN response has been well documented (Chen et al., 2017). For instance, the NS2 of dengue virus (DENV) and hepatitis C (HCV) inhibits type I IFN induction by blocking TBK1/IRF3 phosphorylation (Kaukinen et al., 2013; Dalrymple et al., 2015). The NS3 of HCV is a dominant negative interactor of TBK1 and thus blocks IRF3 activation (Otsuka et al., 2005); it also cleaves MAVS and inhibits RLR-mediated immune responses together with NS2B (Li et al., 2005; Meylan et al., 2005). The NS2A of Kunjin virus (KUNV) inhibits the IFN-induced gene expression (Liu et al., 2004). The NS4A and NS4B of several flaviviruses inhibit JAK-STAT and RLR signaling through multiple mechanisms (Munoz-Jordan et al., 2003; Ding et al., 2013; Nitta et al., 2013; Yi et al., 2015). The NS5 of several flaviviruses including ZIKV are able to interfere with the JAK-STAT signaling and induction of antiviral effectors (Best et al., 2005; Lin et al., 2006; Werme et al., 2008; Ashour et al., 2010; Laurent-Rolle et al., 2010, 2014; Kumthip et al., 2012; Grant et al., 2016). However, to date, there is little information about the molecular mechanisms of immune evasion by ZIKV. We hereby report that ZIKV interferes with the RLR signaling to dampen type I IFN response and enhance its pathogenesis. Our results identify ZIKV NS4A in particular as a suppressor of the RLR pathway by interrupting RLR-MAVS interaction, preventing induction of type I IFNs and inflammatory responses that contain ZIKV replication.

## Materials and Methods

### Reagents and Cell Lines

The rabbit anti-MDA5 (Cat# 5321), RIG-I (Cat# 3743), Myc-tag (Cat# 2278), and Actin (Cat# 8456) were purchased from Cell Signaling Technology (Danvers, MA, United States). M2 (anti-FLAG) magnetic beads (Cat# A2220), anti-FLAG antibody (Cat# F3165), and 3× FLAG peptide (Cat# F4799) were available at Sigma-Aldrich (St. Louis, MO, United States). Mouse anti-human MAVS (Cat# SC-365333) was a product of Santa Cruz Biotechnology (Santa Cruz, CA, United States). Lipofectamine 2000 (Cat# 11668019) was obtained from Thermo Fisher Scientific. The heavy molecule weight polyinosinic-polycytidylic acid (poly I:C-H), light molecular weight (polyI:C-L), and HEK293 cell line stably expressing human TLR4-MD2-CD14 (Cat# 293-htlr4md2cd14) were purchased from InvivoGen (San Diego, CA, United States). The Dual-Luciferase Reporter Assay (Cat# E1910) was available from Promega (Madison, WI, United States). Human embryonic kidney (HEK) 293 T (Cat# CRL-3216), Vero cells (monkey kidney epithelial cells, Cat# CCL-81), placental trophoblast (Cat# CRL-3271) cell lines, and the Zika virus FLR strain (Cat# VR-1844) were from American Type Tissue Culture (Manassas, VA, United States). The Zika virus FS13025 (Cambodia, 2010) was a kind gift of Dr. Tian Wang at the University of Texas Medical Branch at Galveston, TX, United States. Green fluorescence protein (GFP) tagged vesicular stomatitis virus (VSV) was derived from the Indiana strain of VSV (You et al., 2013).

### Plasmid Construction

The DNA encoding the amino residues 1–100, 1–300 together with OMP25 residues 109–145 was cloned into pcDNA3.1-Zeo-N-FLAG vector for expression of mitochondrial FLAG-MAVS1–100, 1–300. The FLAG tagged full-length human MAVS, MAVS Δ101–452, MDA5, RIG-I, ΔRIG-I, GFP-IRF3-5D, and GFP-TBK1 have been described in our previous study (Wang et al., 2013).

The ZIKV FS13025 (Cambodia, 2010, GenBank Accession: JN860885.1) genes (capsid, membrane, envelope, NS1, NS2A, NS2B, NS3, NS4A, and NS4B) were amplified by PCR and inserted into a pcDNA3.1-FLAG or pcDNA3.1-Myc (N-terminal) expression vector. The primers are listed in **Table 1**.

**Table 1:** The ZIKV FS13025 (Cambodia, 2010, GenBank Accession: JN860885.1) gene primers.

ZIKV gene	Primers
Capsid	Forward: GCGGCCGCGAAAAACCCAAAGAAGAAATCCGGA
	Reverse: GGATCCCTATGCCATGGCTGTGGTCAGCA
Membrane	Forward: GCGGCCGCGGTGGAGGTCACTAGACGT
	Reverse: GGATCCCTAGCTGTATGCCGGGGCAATCA
Envelope	Forward: GCGGCCGCGATCAGGTGCATAGGAGTCAG
	Reverse: GAATTCCTATGTGGATAAGAAGATCAA
NS1	Forward: GCGGCCGCGGCCGTCTCTGCTGATGTGG
	Reverse: GGATCCCTACACCATTGACCTTACTAAG
NS2A	Forward: GCGGCCGCGACTGCAGGATCAACTGATCA
	Reverse: GGATCCCTA CCTTGTGAGCAACAGCAGT
NS2B	Forward: GCGGCCGCGAGTGGGAAGCGGAGCTGGC
	Reverse: GGATCCCTACTTCACATACACGTACCA
NS3	Forward: GCGGCCGCGACTGGAAAAAGGAGTGGT
	Reverse: GAATTCCTAGGCAAACTCTTTGAATGAC
NS4A	Forward: GCGGCCGCG GCTGGGAAAAGAGGAGCG
	Reverse: GGA TCCCTA AAGACCCACTGCTACCAT
NS4B	Forward: GCGGCCGCGCTGGGCTTGATTACCGCCA
	Reverse: GGATCCCTA CAAGCCAGCGTTTCTTGTT

### Cell Culture and Transfection

2fGTH and Vero/HEK293T cells and trophoblasts were grown in DMEM/RPMI1640, respectively (Life Technologies, Grand Island, NY, United States) supplemented with 10% FBS and 1× antibiotics/antimycotics in a 37°C incubator filled with 5% CO_2_. Viruses were amplified in Vero cells and the viral titer was determined by plaque forming assays as previously described (Wang et al., 2008). Briefly, 100 μl of viral samples diluted with sterile PBS by 10^1^–10^5^ time were applied to confluent Vero cell monolayer. Plaques were visualized using Neutral red (Sigma-Aldrich) after 1–3 days of incubation at 37°C 5% CO_2_.

#### In-well Transfection

Transfection of plasmid DNA or polyI:C into HEK293T cells with Lipofectamine 2000 (Thermo Fisher Cat# 11668019) was performed following exactly the product manual. Briefly, HEK293T cells were grown in a 24-well culture plate to ∼70–80% confluence on the day of transfection. The plasmid DNA/polyI:C was suspended in 50 μl of serum-free culture medium, and 2 μl of Lipofectamine 2000 in 50 μl of serum-free culture medium. The diluted DNA and Lipofectamine were incubated separately at room temperature for 5 min, and then mixed together. After 15 min incubation, the mix was added to each culture well dropwise with gentle shaking. The transfected cells were incubated at 37°C, 5% CO_2_ for 24 h or desirable time specified in each figure legend.

#### In-suspension Transfection

For hard-to-transfect adherent cells such as trophoblasts, transfection was carried out in cell suspension. Briefly, cells were dislodged by trypsin digestion and 2 × 10^5^ cells were pelleted by brief centrifugation. The cell pellet was then suspended in the transfection mix (DNA + Lipofectamine 2000 prepared as above) for 20 min with intermittent gentle agitation. 0.5 ml of pre-warmed RPMI1640 complete medium was then added and plated into one well of a 24-well plate for further culture.

### Dual Luciferase Reporter Assay

In a 24-well culture plate, 70% confluent HEK293T cells were transfected with 50 ng of pRL-TK reporter (herpes simplex virus thymidine kinase promoter driven renilla luciferase; internal control), 100 ng of ISRE-luciferase reporter (firefly luciferase; experimental reporter) plasmid, and 100 ng of each Zika gene expression plasmid using Lipofectamine 2000. The cells were incubated at 37°C, 5% CO_2_ for 24 h, and then transfected with 10 μg/ml of polyI:C which induces cellular RLR signaling and subsequent IFN-I production. The luciferase activity was measured 16 h after polyI:C stimulation using a Promega Dual Glow kit according to the manufacturer’s instructions.

### Assay for Type I IFN Bioavailability

To quantify Type I IFNs in the cell culture medium of HEK293T, a 2fGTH cell line stably expressing an ISRE-luciferase reporter was used. This cell line responds to type I IFNs and activates an ISRE-driven luciferase reporter (Wang et al., 2013). Briefly 3 × 10^5^ 2fGTH cells/well were seeded and grown in DMEM supplemented with 10% FBS and antibiotics/antimycotics in a 24-well culture plate at 37°C, 5% CO_2_ overnight. Two hundred microliters of pre-stimulated HEK293T culture medium was added to the 2fGTH-ISRE-Luc cells and incubated at 37°C, 5% CO_2_ for 10 h. A serial twofold dilution of recombinant human IFN-β served as positive controls and was used for plotting standard curve. Unstimulated HEK293T cell culture medium served as a negative control.

### Biochemical Assays

For immunoblotting, cells were lysed in lysis buffer [50 mM Tris–HCl (pH 7.5), 150 mM NaCl, 5 mM EDTA, 1% Triton X-100] supplemented with Complete Protease Inhibitor Cocktail (Roche Diagnostics, Indianapolis, IN, United States). Cell lysates were resolved on a 10 or 15% SDS-PAGE gel, and the separated proteins were transferred to a nitrocellulose membrane. The proteins of interest on the membrane were probed with specific primary antibodies, followed by HRP-conjugated secondary antisera, and detected with an enhanced chemiluminescence system from GE Healthcare (Port Washington, NY, United States).

FLAG-immunoprecipitation was performed essentially according to the manufacturer’s manual (Sigma-Aldrich, St. Louis, MO, United States). Briefly, HEK293T cells were transfected with expression plasmids using Lipofectamine 2000. At 24 h post-transfection, the cells were lyzed in lysis buffer (50 mM Tris-HCl, pH 7.4, 0.5% NP-40, 2 mM EDTA, and 150 mM NaCl, with complete protease inhibitors). The lysates were cleared by centrifugation at high speed and then incubated for 2 h with M2 magnetic beads with gentle agitation at 4°C. After 4 washes with ice-cold lysis buffer and 2 washes with 1× Tris buffered saline (TBS), the proteins bound to M2 beads were eluted using 3× FLAG peptides, resolved by SDS-PAGE and detected by immunoblotting.

### Immunofluorescent Microscopy

Trophoblasts were transfected with empty vector or pcDNA3.1-FLAG-NS4A at a DNA/cell ratio of 1 μg/million cells as described in the *In-suspension transfection* section above. The cells were then seeded into an 8-chamber culture slide at a density of 2 × 10^4^ cells per well and incubated overnight at 37°C in 5% CO_2_. The cells were then transfected with 10 μg/ml polyI:C-H as described in the *In-well transfection* section above. Eight hours after polyI:C-H treatment, trophoblasts were then washed with ice-cold 1× PBS once, fixed with 4% paraformaldehyde (PFA) for 30 min at room temperature. After brief wash, the cells were then perforated with 0.5% triton X-100 for 15 min, and blocked with 4% goat serum for 30 min at room temperature. The cells were incubated with appropriated diluted primary antibodies at 4°C overnight with gentle agitation. After 3 washes with 1× PBS, the cells were incubated with Alexa Fluor 594/488-conjugated secondary antibodies and 4′,6-diamidino-2-phenylindole (DAPI) for 1 h at room temperature with gentle agitation. Fluorescent images were acquired using NIS-Elements Imaging Software on an inverted Nikon Eclipse Ti fluorescence microscope.

### Generation of Gene Knockout With the CRISPR-Cas9 Technology

The gene specific guide RNA was cloned into lentiCRISPRv2 vector and co-transfected into HEK293T cells with the packaging plasmids pVSV-G and psPAX2 (Sanjana et al., 2014; Shalem et al., 2014). Forty-eight hours after transfection, the lentiviral particles in the cell culture media were applied to trophoblasts for 48 h. The transduced cells were then selected with puromycin at 1 μg/ml for 48–72 h. Successful knockout clones were confirmed by immunoblotting. The guide RNA for RIG-I, MDA5, and MAVS was TCCTGAGCTACATGGCCCCC, CTTTCTGCCTGCAGAGGTGA, and AAGTTACCCCATGCCTGTCC, respectively. The wild type control was lentiCRISPRv2 vector only.

### Reverse Transcription and Quantitative PCR (qPCR)

Cells were collected in 350 μl of RLT buffer (QIAGEN RNeasy mini kit). RNA was extracted following the QIAGEN manual exactly, reverse-transcribed into cDNA using the BIO-RAD iScript^TM^ cDNA Synthesis Kit. qPCR was performed with gene-specific primers and SYBR Green master mix. Results were calculated using the -ΔΔCt method and beta actin gene as an internal control. The qPCR primers and probes for immune genes were reported in our previous studies (Wang et al., 2010, 2012). The primer pair for ZIKA virus was 5′ CCGCTGCCCAACACAAG-3′ and 5′ CCACTAACGTTCTTTTGCAGACAT-3′.

### Graphing and Statistics

For all the bar graphs, data were expressed as mean ± SEM. A standard two-tailed unpaired Student’s *t*-test was used for statistical analysis. The results with a *p*-value ≤ 0.05 were considered significant.

## Results

### Identification of ZIKV Genes That Interfere With the RLR Pathway

**FIGURE 1:**
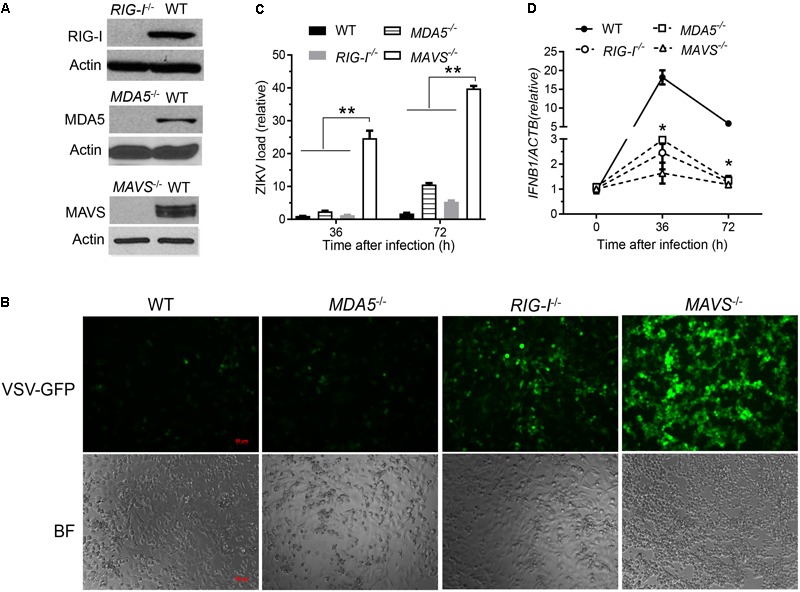
The RLR signaling is essential for induction of type I IFN responses to and control of ZIKV infection in human trophoblasts. **(A)** Immunoblots of RIG-I, MDA5, MAVS and a house keeping gene actin protein expression in wild type (WT) and knockout human trophoblasts. **(B)** Fluorescent microscopic images of GFP in trophoblasts infected with VSV-GFP at a multiplicity of infection (MOI) of 0.1 for 20 h. Objective: 5×, scale bar: 50 μM. qPCR quantification of cellular **(C)** viral RNA and **(D)** *IFNB1* mRNA in trophoblasts infected with ZIKV FLR at a MOI of 5 for the indicated time. The bars/data points in **C,D** are the mean + SEM of the results, *n* = 3. ^∗^*p* < 0.0.5; ^∗∗^*p* < 0.01 (Student’s *t*-test). The data shown are representative of three independent experiments.

**FIGURE 2:**
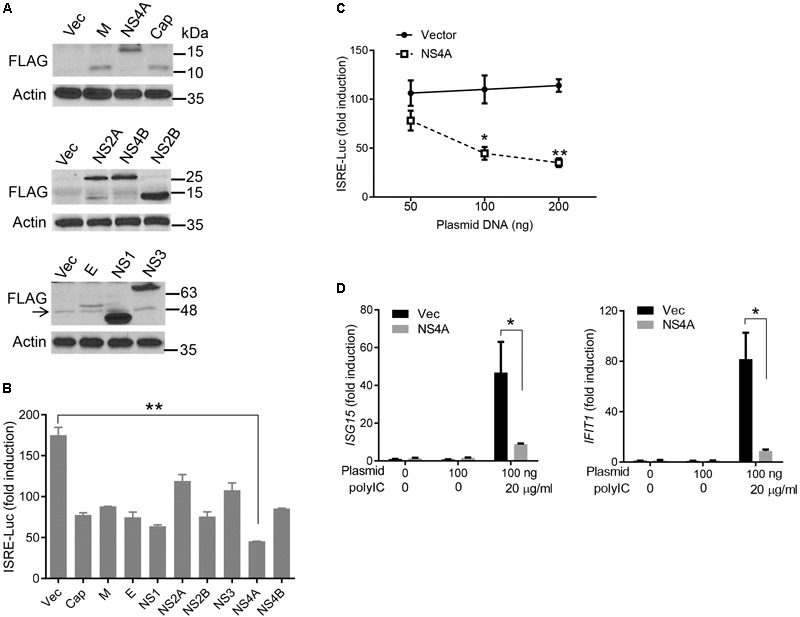
NS4A inhibits polyI:C- induced type I IFN responses. **(A)** Immunoblots of the FLAG-tagged ZIKV protein expression in HEK293T cells. The proteins were detected by immunoblotting with an anti-FLAG antibody. Actin is a housekeeping protein control. The arrow indicates a non-specific band. The molecular weights in kDa are marked to the right of the blots. **(B)** Quantification of ISRE-Luc activity in HEK293T cells transfected with either 100 ng of empty vector or the indicated ZIKV gene expressing plasmid for 24 h and then stimulated with 10 μg/ml of heavy molecular weight polyI:C-H for 16 h. Data are expressed as fold induction over unstimulated cells. **(C)** Quantification of ISRE-Luc activity by a dual luciferase reporter assay similar to **B** except that an increasing dose of vector and FLAG-NS4A plasmids was applied. **(D)** Quantification of *ISG15* and *IFIT1* mRNA by q-PCR in HEK293T cells transfected with either 100 ng of empty vector or NS4A, and then stimulated with polyI:C as in **B**. The bars/data points in **B–D** represent the mean + SEM of the results, *n* = 3. ^∗^*p* < 0.0.5; ^∗∗^p < 0.01 (Student’s *t*-test). The data shown are representative of three independent experiments.

**FIGURE 3:**
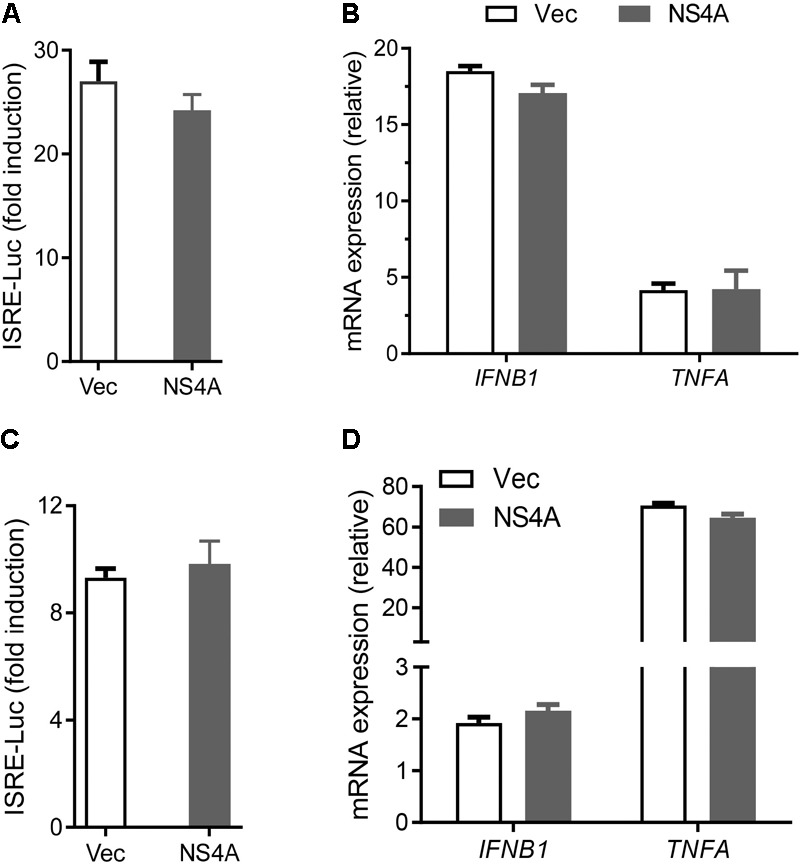
NS4A does not interfere with TLR3 or TLR4 signaling. **(A)** Quantification of ISRE-Luc activity in HEK293T cells transfected with Myc vector + FLAG-TLR3 or Myc-NS4A + FLAG-TLR3 (100 ng each) for 16 h and then stimulated with 20 μg/ml low molecular weight polyI:C-L (no transfection) for 24 h. **(B)** Quantification of *IFNB1* and *TNFA* mRNA by qPCR. The cells were treated similarly as in **A** except that polyI:C stimulation was 8 h. **(C)** Quantification of ISRE-Luc activity in stable TLR4/MD2/CD14-expressing HEK293 cells transfected with 100 ng of Myc vector or Myc-NS4A plasmid for 12 h and stimulated with 100 ng/ml of LPS for 24 h. **(D)** Quantification of *IFNB1* and *TNFA* mRNA by qPCR. The cells were treated similarly as in **C** except that LPS stimulation was 8 h. The results are expressed as fold change over unstimulated cells. The bars represent the mean + SEM of the results, *n* = 3. Three biological replicates were pooled for qPCR. ^∗^*p* < 0.0.5; ^∗∗^*p* < 0.01 (Student’s *t*-test). The data shown are representative of three independent experiments.

The cytosolic RLRs recognize viral dsRNA intermediates and induces type I IFNs to control viral replication. We first asked if this signaling pathway was also important for anti-ZIKV responses in human trophoblast, a cell type of the placental barrier that is physiologically relevant to ZIKV congenital transmission (Miner et al., 2016). We first successfully abolished MDA5, RIG-I, and MAVS protein expression using the CRISPR-Cas9 system (**Figure 1A**), and validated their phenotype using vesicular stomatitis virus (VSV) which is predominantly sensed by RIG-I. Indeed, the viral load was the same in *MDA5*^-/-^ as WT, but much higher in *RIG-I*^-/-^ and *MAVS*^/-^ than WT cells (**Figure 1B**). We next assessed ZIKV infection and type I IFN expression in these cells. The abundance of intracellular ZIKV RNA was modestly higher in *RIG*^-/-^ and *MDA5*^-/-^ than wild type (WT) cells at 36 and 72 h after infection, while it was dramatically increased by ∼20–40 times in *MAVS*^-/-^ compared to WT cells (**Figure 1C**). The *IFNB1* mRNA induction was reduced by ∼7-fold in *RIG*^-/-^ and *MDA5*^-/-^, and almost completely abolished in *MAVS*^-/-^ compared to WT cells (**Figure 1D**). These results clearly demonstrate an essential role for the RLR signaling in type I IFN induction and resisting ZIKV infection in human trophoblasts. Next, we screened 9 individual ZIKV proteins to assess whether any would dampen type I IFN response. We subcloned the genes of ZIKV FS13025 strain into a pcDNA3.1-FLAG vector and transfected these plasmids into HEK293T cells, an established cell line for study of functional RLR signaling. We confirmed each ZIKV protein expression by Western blotting using an anti-FLAG antibody (**Figure 2A**). Then we assessed the impact of each ZIKV protein on type I IFN response which is rapidly induced by viral infection and critical for controlling initial viral replication. We used an ISRE-driven luciferase reporter system to determine type I IFN induction by polyI:C, a synthetic double-stranded RNA that activates the RLR signaling. We noted that all the ZIKV proteins reduced ISRE-Luc to variable degrees compared to the vector control, with the most dramatic reduction seen with NS4A (**Figure 2B**). We hereby focused on NS4A and validated its inhibitory function using 3 different doses of NS4A plasmid DNA. We noted that NS4A repressed polyI:C-induced ISRE-Luc activity in a dose-dependent manner (**Figure 2C**). Consistently, the mRNA expression of representative interferon stimulated genes (ISG) *ISG15* and *IFIT1* was also repressed significantly by NS4A (**Figure 2D**). These data suggest that NS4A interferes with the RLR signaling. We next tested if NS4A influenced other virus-sensing pathways such as the Toll-like receptors (TLRs). Because HEK293T cell line expresses little TLR3, a modified TLR3 that localizes to the plasma membrane (Wang et al., 2013) together with the empty vector or NS4A was transiently expressed and its signaling was stimulated with non-transfected polyI:C (to avoid stimulation of intracellular RLRs). As shown in **Figure 3A**, the ISRE-Luc induction was not affected by NS4A compared to the vector control. Consistently, the *IFNB1* and *TNFA* mRNA expression was the same between the vector and NS4A (**Figure 3B**). We observed a similar result with the TLR4 signaling pathway (**Figures 3C,D**). These results indicate that NS4A does not interfere with TLR signaling.

### NS4A Suppresses RIG-I and MDA5-Induced Type I Interferons

**FIGURE 4:**
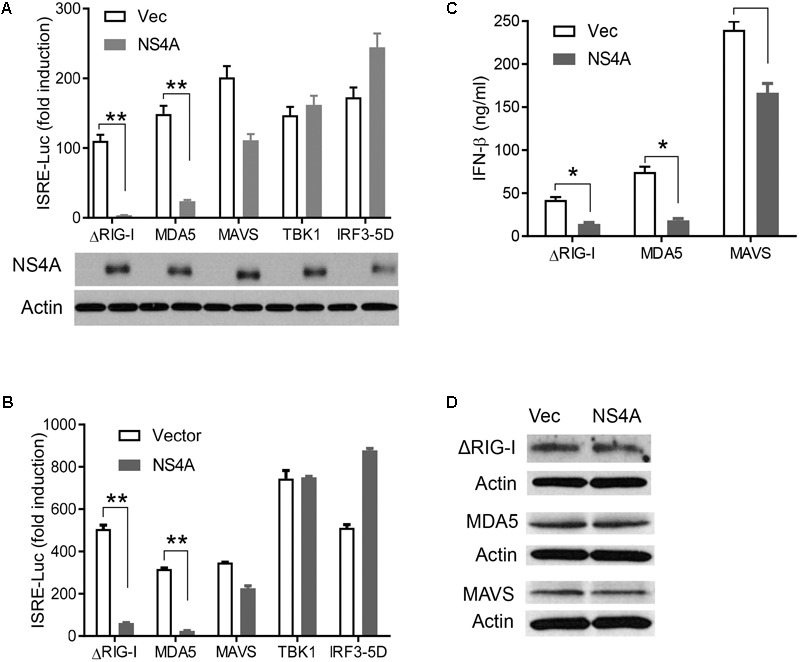
NS4A interferes with RIG-I and MDA5-induced type I interferons. Quantification of ISRE-Luc activity in HEK293T cells transfected with 100 ng of FLAG vector or FLAG-NS4A of **A** ZIKV FS13025 and **B** FLR strain, together with the indicated individual immune genes of the RLR pathway for 24 h. The immunoblots under the bar graph show the FLAG-NS4A and actin protein expression. **(C)** Quantification of type I IFN bioavailability in the culture media of **A**. **(D)** Immunoblots of FLAG-ΔRIG-I, MDA5, and MAVS proteins in HEK293T cells treated exactly as in **A**. The FLAG-tagged protein was detected with an anti-FLAG antibody. The bars in **A–C** represent the mean + SEM of the results, *n* = 3. ^∗^*p* < 0.0.5; ^∗∗^*p* < 0.01 (Student’s *t*-test). The data shown are representative of three independent experiments.

The aforementioned results demonstrate that NS4A interferes with the RLR pathway and prompt us to pinpoint the exact step that NS4A targets. We expressed NS4A concurrently with ΔRIG-I, MDA5, MAVS, TBK1, or IRF3-5D, the key components that can induce ISRE-Luc when transiently overexpressed (Wang et al., 2013). NS4A repressed both RIG-I- and MDA5-induced ISRE-Luc activity significantly and had a modest effect on MAVS-mediated ISRE-Luc induction (**Figure 4A**). However, NS4A did not significantly affect TBK1 or IRF3-induced ISRE activity (**Figure 4A**). Given the possibility of strain-dependent variations in ZIKV immune evasion, we tested NS4A of a recent South American strain FLR 2015 and noted a similar result (**Figure 4B**), suggesting the immune evasion capacity of NS4A is conserved during ZIKV evolution. In addition to quantification of intracellular interferon promoter activity with an ISRE reporter system, we conducted a bioassay to measure the concentrations of secreted type I IFN proteins (mainly IFN-β) by HEK293T. NS4A reduced both RIG-I and MDA5-induced IFN-β protein expression significantly compared to vector control (**Figure 4C**). Ectopic expression of a protein may squelch concurrent expression of another protein non-specifically. In our experimental conditions, NS4A did not impair ΔRIG-I, MDA, or MAVS expression (**Figure 4D**). These results suggest that NS4A targets the upstream of the RLR pathway, e.g., RIG-I and MDA5 directly.

### NS4A Interacts With MAVS

**FIGURE 5:**
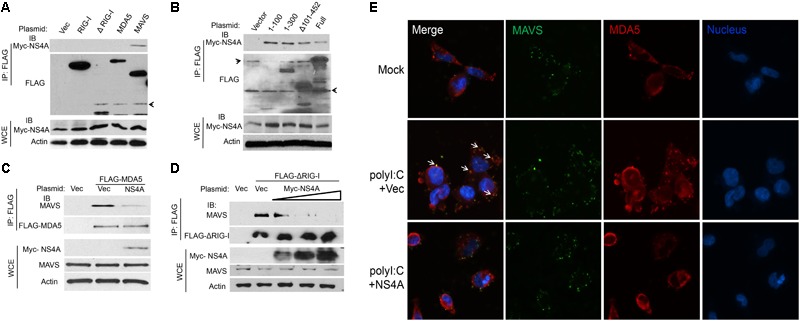
NS4A interacts with MAVS and blocks RLR binding to MAVS. **(A)** Co-immunoprecipitation (co-IP) of MAVS with NS4A from HEK293T cells transfected with Myc-NS4A and the indicated FLAG-tagged genes using an anti-FLAG monoclonal antibody, followed by immunoblotting (IB). WCE, whole-cell extract. **(B)** co-IP of the truncated forms of MAVS with NS4A from HEK293T cells transfected with Myc-NS4A and FLAG-tagged MAVS mutants using an anti-FLAG monoclonal antibody. co-IP of **(C)** FLAG-MDA5 or **(D)** FLAG-ΔRIG-I with endogenous MAVS from HEK293T cells transfected with FLAG-MDA5 or FLAG-ΔRIG-I and Myc vector or increasing amounts of Myc-NS4A. The plasmid DNA molar ratio of Myc- NS4A to FLAG-MDA5 is 1:3; to FLAG-ΔRIG-I are 1:6, 1:3, and 1:2. **(E)** Immunofluorescent staining of endogenous MAVS and MDA5 in human trophoblasts. Trophoblasts were untreated (Mock) or transfected with either a FLAG vector or FLAG-NS4A for 24 h and then stimulated with 20 μg/ml of heavy molecular weight polyI:C-H for 8 h. MDA5/MAVS was stained with a rabbit anti-MDA5 and mouse anti-MAVS antibody followed by secondary antibodies conjugated with Alexa Fluor 594/488. The nuclei were counter-stained with DAPI. The images were acquired using an inverted Nikon Eclipse Ti fluorescence microscope. The arrows indicate colocalizations of MDA5 and MAVS. In **(A–C)** actin is a housekeeping protein control. The arrow heads point to non-specific bands. The data shown are representative of three independent experiments.

The aforementioned results suggest that NS4A interferes with RLR function directly; we then asked if it is a dominant negative interactor of RLRs. We co-expressed Myc-NS4A together with FLAG tagged RIG-I, MDA5, MAVS, or ΔRIG-I (only the two CARDs domain mediating binding to MAVS) respectively, and then performed a co-immunoprecipitation assay using an anti-FLAG antibody. We observed that only FLAG-MAVS, but surprisingly not RIG-I, MDA5, or ΔRIG-I, co-immunoprecipitated with Myc-NS4A (**Figure 5A**). It has been well established that the N-terminal CARD domain of MDA5/RIG-I interacts with the N-terminal CARD of MAVS to activate recruitment of downstream TRAF3/6 to its C-terminus. We asked if NS4A competes with MDA5/RIG-I for binding to the CARD of MAVS. Indeed, NS4A co-precipitated with the CARD-containing truncate of MAVS, most strongly with the CARD only (1–100) (**Figure 5B**). Furthermore, NS4A expression reduced the amount of endogenous MAVS bound by FLAG-MDA5 (**Figure 5C**) and FLAG-ΔRIG-I in a dose-dependent manner (**Figure 5D**). By immunofluorescent microscopy, we observed that endogenous MAVS colocalized with MDA5 very well in polyI:C-stimulated cells (polyI:C+Vec) when compared to unstimulated cells (Mock). The co-localizations between MAVS and MDA5, however, were significantly reduced in the presence of NS4A (polyI:C+NS4A) (**Figure 5E**). These results indicate that NS4A occupies the CARD of MAVS to block MDA5/RIG-I access.

## Discussion

The relative contribution of different classes of pathogen pattern recognition receptors (PRRs) to innate antiviral immune responses may vary with viral species and tissue cell types. A previous study revealed that the cytoplasmic PRR, MDA5, and RIG-I, recognized a specific subset of RNA viruses (Kato et al., 2006). However, both MDA5 and RIG-I play a non-redundant additive role in type I IFN induction by flaviviruses (Wilkins and Gale, 2010). A recent study demonstrated that TLR3, MDA5, and RIG-I all play a moderate role in limiting ZIKV infection in primary human skin fibroblasts (Hamel et al., 2015). Our results, however, reveal that the RLR pathway is essential for inducing type I IFN responses to ZIKV infection in human trophoblasts. Knockout of either RIG-I or MDA5 reduced, while deletion of the common adaptor for both RIG-I and MDA5, MAVS, almost completely abolished type I IFN induction and supports productive ZIKV infection. Similar results have been previously reported for a sibling of ZIKV, West Nile virus (WNV) studies (Fredericksen et al., 2008; Suthar et al., 2010). Our results suggest that MDA5 and RIG-I each contribute to a significant amount of anti-ZIKV type I IFN responses. RIG-I may rapidly initiate early; while MDA5 maintains late innate immune responses to WNV (Errett et al., 2013). This sequential, non-redundant function of RIG-I and MDA5 ensures an effective immune response throughout the course of viral infection. Understanding the major antiviral pathways in trophoblasts is physiologically meaningful as these cells form a barrier to ZIKV congenital transmission and prevent pathogenesis of microcephaly. Given its essential role in induction of anti-ZIKV immune responses in trophoblasts, stimulation of the RLR signaling may thus be potentially prophylactic and therapeutic against ZIKV complications. Indeed, as proof-of-principle, RIG-I agonists have been recently shown to potently restrict ZIKV infection in human dendritic cells (Bowen et al., 2017).

It has been established that flaviviruses have evolved complex mechanisms to avoid the host immune responses including the RLR signaling [reviewed by Asif et al. (2017) and Chen et al. (2017)]. As a new re-emerging flavivirus, ZIKV has recently attracted much public attention for its potential association with neonatal microcephaly and adult Guillain-Barre Syndrome (Asif et al., 2017). Its pathogenesis, however, remains largely obscure. ZIKV NS5 was recently shown to induce human STAT2 degradation (Grant et al., 2016); NS4A and NS4B inhibit neurogenesis via Akt-mTOR signaling and induce autophagy (Liang et al., 2016). In this study, we observed that almost all the ZIKV protein overexpression down-regulated polyI:C- induced type I IFN response, with NS4A being the most potent. We further found that NS4A repressed the RLR signaling by targeting MAVS. Our conclusions are supported by several lines of evidence. First, NS4A dramatically repressed type I IFN induction by ectopic overexpression of MDA5 and RIG-I, but not MAVS, TBK1, or IRF3. Second, NS4A interacted with the N-terminal CARD of MAVS but not RIG-I or MDA5, and competed with MDA5/RIG-I for binding to MAVS. However, binding of NS4A to MAVS did not impair IFN-I induction by MAVS overexpression. These results seem contradictory. But the mechanisms of MAVS activation are different under overexpression or viral infection/polyI:C stimulation conditions. When overexpressed, MAVS undergoes self-oligomerization and recruits downstream TRAF3/6 to its C-terminus independently of upstream RIG-I/MDA5. Thus NS4A binding to the N-terminal CARD of MAVS does not influence its downstream signaling events. In the ZIKV infection/polyI:C stimulation conditions, MAVS needs to be activated by RIG-I/MDA5 binding to its CARD domain, which can be blocked by NS4A. The mode of ZIKV NS4A action is similar to that of dengue virus NS4A which disrupts RLR-MAVS interaction and also MAVS signaling (He et al., 2016). Since both RIG-I and MDA5 contribute to the type I IFN responses to ZIKV infection, blocking MAVS is more energy-efficient than inhibiting individual RIG-I and MDA5 separately.

The ISRE luciferase reporter system reflects both primary and secondary type I IFN response, as ISRE can be activated by both IRF3/7 and STAT1/2. However, NS4A specifically inhibits RLRs-, but not their downstream factors like MAVS/TBK1/IRF3-induced ISRE reporter activity. These results suggest that NS4A does not target the JAK-STAT pathway.

In addition to a role for ZIKV NS4A in antagonizing RLR signaling, NS4A has been recently discovered to interact with NS4B and induce cellular autophagy in human neural stem cells by interfering with the Akt-mTOR signaling, leading to defective neurogenesis characteristic of microcephaly (Liang et al., 2016). Induction of autophagy by NS4A/4B may also amplify ZIKV replication (Cao et al., 2017). Thus, NS4A may be potentially involved in both immune evasion and abnormal brain development, two key aspects of ZIKV pathogenesis. The multidimensional pathogenic features of NS4A make it a potential therapeutic target.

## Author Contributions

JM performed the majority of the experimental procedures. HK, TG, EL, LW, and JX contributed to some of the results and provided technical support. QS, ZZ, YC, and LY contributed to data analysis and provided technical support. PW conceived, designed the studies, and wrote the paper. All the authors reviewed and modified the manuscript.

## Conflict of Interest Statement

The authors declare that the research was conducted in the absence of any commercial or financial relationships that could be construed as a potential conflict of interest.
